# Endoscopic resection of an extraluminal esophageal duplication cyst

**DOI:** 10.1055/a-2638-6080

**Published:** 2025-07-15

**Authors:** Shaimaa Elkholy, Mohamed El-Sherbiny, Hussein Hassan Okasha, Abeer Abdallatef, Hany Haggag, Mohamed Abdel Zaher, Karim Essam

**Affiliations:** 1 63527 Gastroenterology Division, Internal Medicine Department, Cairo University Kasr Alainy Faculty of Medicine, Cairo, Egypt; 2 63527 MBBCh, Cairo University Kasr Alainy Faculty of Medicine, Cairo, Egypt

Esophageal duplication cysts are rare congenital anomalies, occasionally presenting in adults with dysphagia 1 . A 32-year-old man presented with progressive dysphagia. Upper endoscopy revealed a subepithelial bulge in the distal esophagus ( Fig. 1 ). A computed tomography (CT) scan showed a well-defined, homogeneous soft-tissue mass causing extrinsic compression of the esophageal lumen. The lesion was initially interpreted radiologically as an esophageal leiomyoma ( Fig. 2 **a** ). Endoscopic ultrasound demonstrated a well-defined cystic lesion (25 × 47 mm) at 35 cm from the dental arch, with posterior acoustic enhancement, no mural nodules or vascularity, and multiple wall layers containing echogenic content, suggestive of a duplication cyst 1 2 ( Fig. 3 ).

**Fig. 1:**
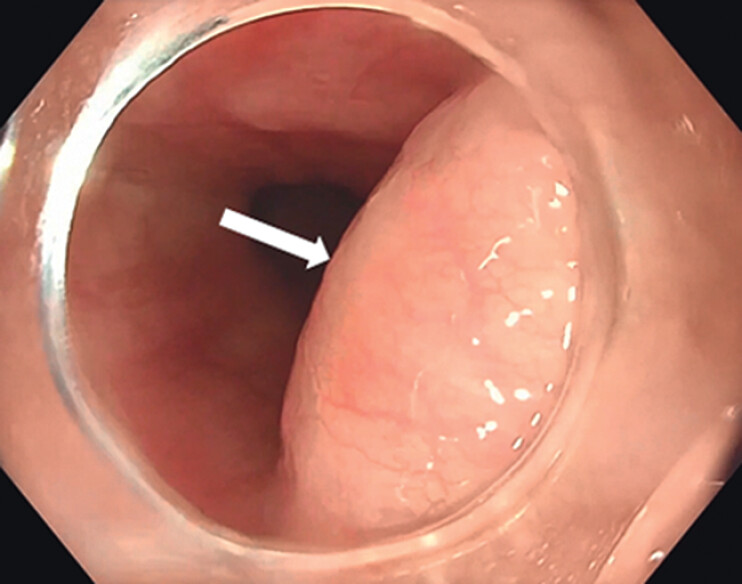
Upper endoscopy showing a subepithelial bulge (white arrow) in the lower esophagus.

**Fig. 2:**
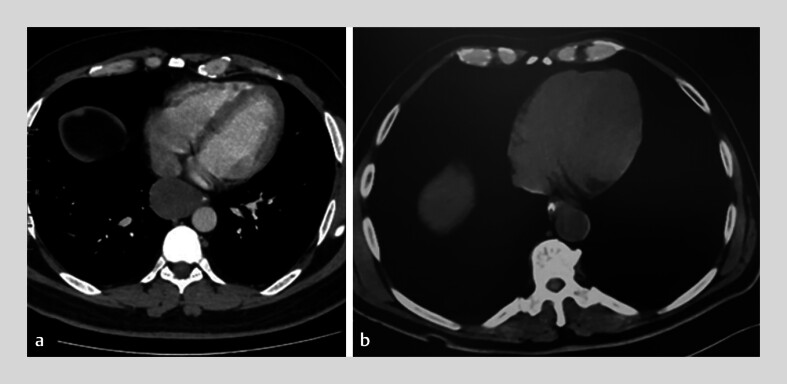
**a** Axial contrast-enhanced computed tomography (CT) scan showing a well-defined, homogeneous soft-tissue mass causing extrinsic compression of the esophageal lumen. The lesion was initially interpreted radiologically as an esophageal leiomyoma. Subsequent endoscopic and histopathological evaluations confirmed the diagnosis of an esophageal duplication cyst. **b** Follow-up CT scan demonstrating complete resection of the cyst with resolution of the esophageal compression.

**Fig. 3:**
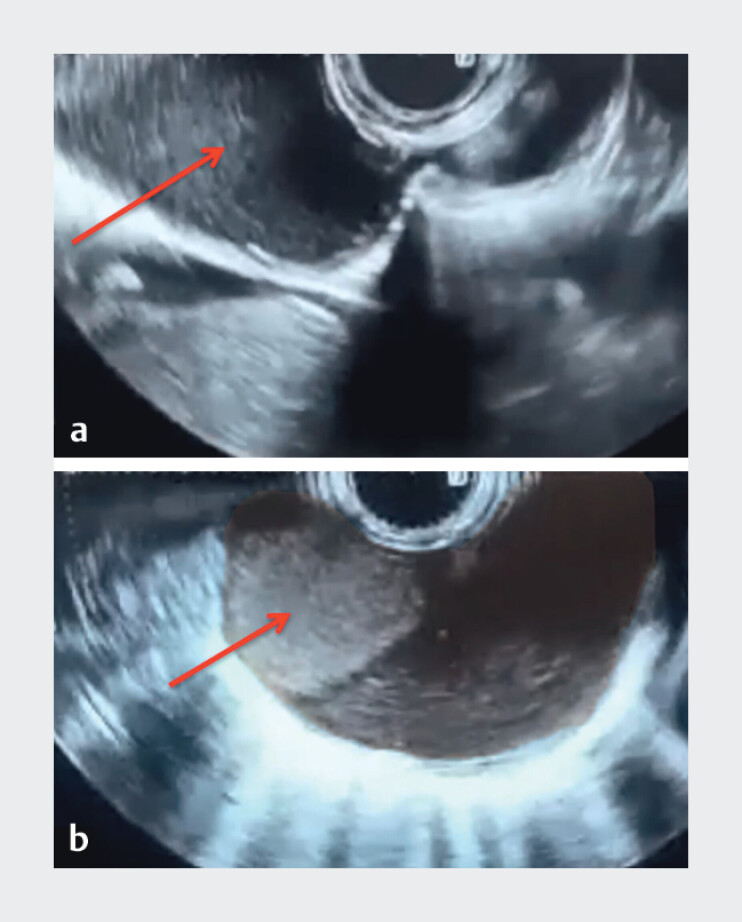
**a** Endoscopic ultrasound (EUS) image demonstrating a well-defined extraluminal cystic lesion (red arrow) causing a bulge in the lower esophageal wall. **b** EUS with color Doppler revealing a cystic lesion with posterior acoustic enhancement and absence of internal vascularity. The red arrow highlights a soft tissue component with a horizontal upper margin and no Doppler flow, suggestive of echogenic fluid layering in the dependent portion – likely representing sludge or proteinaceous material.

After multidisciplinary team consultation, submucosal tunnel endoscopic resection (STER) was selected as the preferred minimally invasive approach 3 . A submucosal tunnel was created following submucosal injection and mucosal incision. As dissection progressed, the cyst was seen bulging into the tunnel lumen ( Video 1 ). Shortly thereafter, the cyst was unintentionally punctured, releasing mucoid contents. Suction was applied, and the cyst wall was completely dissected and removed ( Fig. 4 ). The procedure used an Olympus X1 endoscope (GIF-H1500; Olympus Corp., Tokyo, Japan), a HybridKnife (ERBE, Tübingen, Germany), an ITknife (Olympus Corp., Tokyo, Japan), and an ERBE Vio 3 (Endocut Q 3,3,3; precise SECT coagulation 4.5).

Demonstration of submucosal tunnel endoscopic resection for a symptomatic esophageal duplication cyst in a 32-year-old man and six-month follow-up endoscopy.Video 1

**Fig. 4:**
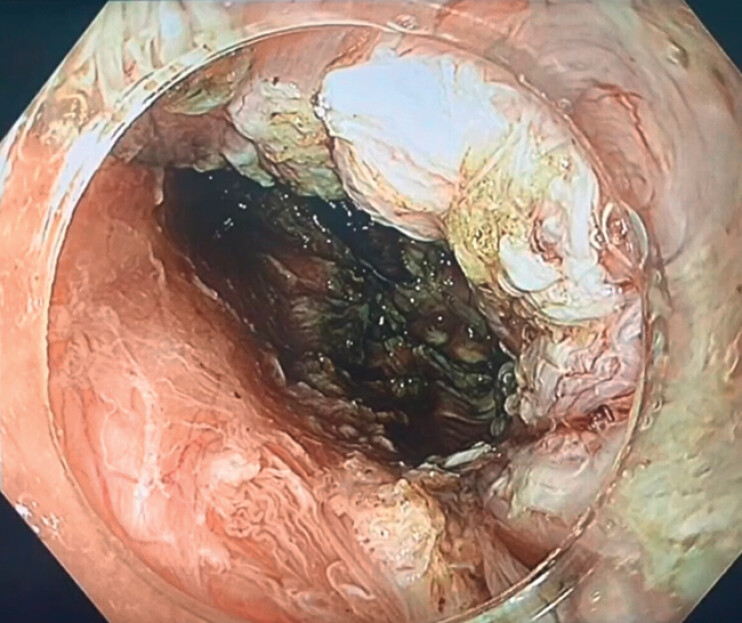
Endoscopic view of the submucosal tunnel bed following complete resection of the cyst.

Histopathology confirmed an esophageal duplication cyst lined with pseudostratified ciliated columnar epithelium and surrounded by smooth muscle layers – consistent with an enteric-type duplication cyst 1 ( Fig. 5 ). Follow-up CT showed complete cyst resection ( Fig. 2 **b** ), and an upper endoscopy after six months revealed a scar at the previous entry site with complete resolution of the esophageal bulge ( Video 1 ).

**Fig. 5:**
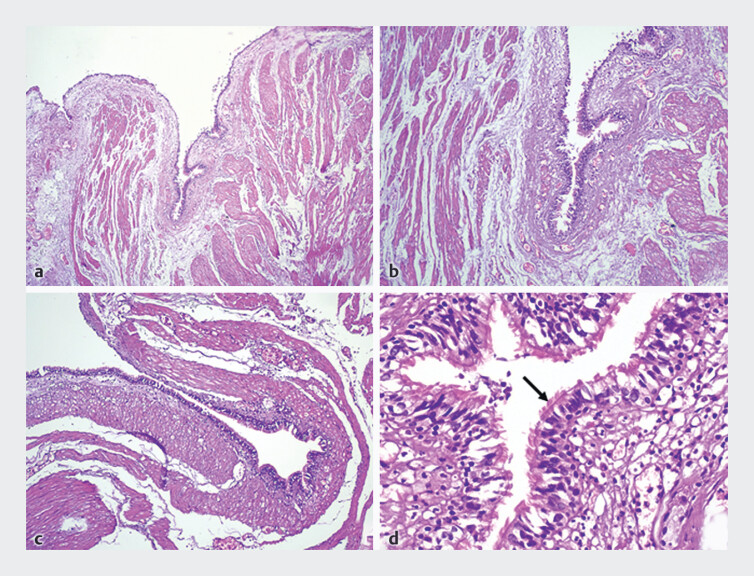
Histopathological examination revealing a cyst wall lined by pseudostratified ciliated columnar epithelium (arrow), overlying a thin fibrous stroma and surrounded by smooth muscle fibers that merge with the muscularis propria. Hematoxylin and eosin (H&E) stain – Original magnifications: ×40, ×40, ×100, and ×400 (respectively).

This case demonstrates the utility of STER as a safe and effective organ-preserving approach for managing benign subepithelial esophageal lesions such as duplication cysts 2 3 4 and underscores the evolving capabilities of endoscopic resection in addressing even extraluminal pathology.

Endoscopy_UCTN_Code_TTT_1AO_2AG_3AZ

## Notes

Endoscopy E-Videos https://eref.thieme.de/e-videos
*E-Videos* is an open access online section of the journal *Endoscopy* , reporting on interesting cases and new techniques in gastroenterological endoscopy. All papers include a high-quality video and are published with a Creative Commons CC-BY license. Endoscopy E-Videos qualify for HINARI discounts and waivers and eligibility is automatically checked during the submission process. We grant 100% waivers to articles whose corresponding authors are based in Group A countries and 50% waivers to those who are based in Group B countries as classified by Research4Life (see: https://www.research4life.org/access/eligibility/ ). This section has its own submission website at https://mc.manuscriptcentral.com/e-videos .
